# Granulopoiesis inhibition in acute inflammation: comparative studies in healthy and leukaemic mice

**DOI:** 10.1155/S0962935192000267

**Published:** 1992

**Authors:** Mohamad Hamood, Francis Corazza, Pierre Francois Bluche, Hassan El Teraifi, Pierre Fondu

**Affiliations:** 1 Experimental Hematology Laboratories of Brugmann University Hospital and Joseph Bracops Hospital Free University of Brussels Place Van Gehuchten 4 Brussels 1020 Belgium; 2 Department of Histopathology Christie Hospital Manchester UK

## Abstract

It was demonstrated previously that mice undergoing an inflammatory reaction induced by subcutaneous (SC) implantation of copper rods, produce humoral factors that initially enhance, but subsequently inhibit, diffusion chamber (DC) granulopoiesis. This provided evidence that granulopoiesis is under the control of both humoral stimulators and inhibitors. In order to test the granulopoietic regulatory mechanism in leukaemic mice, we investigated the regulatory role of granulopoietic humoral inhibitors during in vivo granulopoiesis. We noticed that mice suffering from acute myeloid leukaemia (AML) are unable to augment the production of these humoral inhibitory factors when acute inflammation is induced, since no change in DC cell content was observed with or without prior inflammation. Moreover, unlike healthy mice, the serum of leukaemic mice withdrawn during the inhibition phase of acute inflammation did not show any inhibitory activity toward granulocyte—monocyte (GM) colony growth in vitro. Our results also show that increased levels of normal humoral inhibitors do not influence the proliferation and/or differentiation of leukaemic cells implanted in diffusion chamber cultures.


Mediators of Inflammation, 1, 171–175 (1992)

### Granulopoiesis inhibition in acute inflammation: comparative studies in healthy and leukaemic mice

**Mohamad Hamood,[1,CA] Francis Corazza,[1] Pierre Francois Bluche,[1] Hassan El Teraifi,[2] and Pierre Fondu[1]**

[1] Experimental Hematology Laboratories of Brugmann University Hospital and Joseph Bracops Hospital, Free University of Brussels, Place Van Gehuchten 4, 1020 Brussels, Belgium; [2] Department of Histopathology, Christie Hospital, Manchester, UK

[CA] Corresponding Author

**It was demonstrated previously that mice undergoing an inflammatory reaction induced by subcutaneous (SC) implantation of copper rods, produce humoral factors that initially enhance, but subsequently inhibit, diffusion chamber (DC) granulopoiesis. This provided evidence that granulopoiesis is under the control of both humoral stimulators and inhibitors. In order to test the granulopoietic regulatory mechanism in leukaemic mice, we investigated the regulatory role of granulopoietic humoral inhibitors during *in vivo* granulopoiesis. We noticed that mice suffering from acute myeloid leukaemia (AML) are unable to augment the production of these humoral inhibitory factors when acute inflammation is induced, since no change in DC cell content was observed with or without prior inflammation. Moreover, unlike healthy mice, the serum of leukaemic mice withdrawn during the inhibition phase of acute inflammation did not show any inhibitory activity toward granulocyte—monocyte (GM) colony growth *in vitro*. Our results also show that increased levels of normal humoral inhibitors do not influence the proliferation and/or differentiation of leukaemic cells implanted in diffusion chamber cultures.**

**Key words:** Acute inflammation, Acute myeloid leukaemia (AML), Diffusion chamber (DC), Granulopoiesis, Humoral granulopoietic inhibitor

### Introduction

Control of *in vivo* granulopoiesis by humoral factors is difficult to study for several reasons. Among these reasons are the lack of a satisfactory label for newly released granulocytes, and the emptying of important intramedullary storage pools by injection of foreign proteins. For these reasons, several investigators have used the *in vivo* intraperitoneal diffusion chamber (DC) culture technique.[1] This technique allows proliferation and differentiation of nearly all types of haemopoietic cells, and shows that *in vivo* haemopoietic cell proliferation and differentiation are under the control of humoral regulators.[1] Under states of increased demand, such as acute inflammation, DC granulopoiesis was regulated by both granulopoietic humoral stimulators and inhibitors.[2] When DCs, loaded with a constant number of murine bone marrow cells, are implanted into mice that were stimulated the previous day by an aseptic abscess, significantly more proliferative and non-proliferative granulocytes, as well as their progenitors, develop in DC. However, when DCs are implanted 6 days after the induction of acute inflammation, a significant depression of DC granulopoiesis is observed.[2] This suggests that the release of both humoral granulopoietic stimulators and inhibitors occurs consecutively.

The physiological granulopoietic regulatory mechanism in states of increased demand is not clear in leukaemic subjects, and the failure of normal granulopoiesis in leukaemic hosts is not well understood. However, recent investigations[3] showed that leukaemic mice are unable to overproduce humoral granulopoietic stimulators during the stimulation phase of acute inflammation. The increased levels of these stimulators do not influence either the proliferation or the differentiation of leukaemic cells implanted in DC.

In the present study we investigated the inflammatory response of leukaemic mice during the inhibition phase of acute inflammation, in order to examine the ability of the leukaemic host to produce physiological granulopoietic inhibitors. In addition, we examined the effect of normal humoral granulopoietic inhibitors on leukaemic cell differentiation and proliferation.

### Materials and Methods

*Mice:* Male CBA/H mice (Centre d'animaux de laboratoire, Heverlee, Belgium) 16–18 weeks of age,

© 1992 Rapid Communications of Oxford Ltd were used as host animals for the diffusion chamber cultures and as a source for adult normal and leukaemic bone marrow cells.

*Induction of acute inflammation:* Acute inflammation followed by an aseptic abscess formation was induced by subcutaneous insertion of metallic copper rods under sterile conditions as previously described.[2] A surgical incision was made in the left paravertebral area in control mice (sham operation) to exclude non-specific effects of surgery on haemopoiesis.

*Induction of acute myeloid leukaemia (AML):* Acute myeloid leukaemic cells were obtained from the spleen of CBA/H leukaemic mice (Radiobiology Unit, Harwell, Didcot, Oxon, UK). AML was induced by ionizing radiation[4] and maintained by serial intravenous inoculation into new CBA/H mice as previously described.[5] Each host CBA/H mouse was injected intravenously with 0.2 ml of suspension medium containing one million leukaemic splenic cells, 8 days prior to copper insertion. The suspension medium was Hanks's Balanced Salt Solution (HBSS) with 10% fetal calf serum.

*Diffusion chamber cultures:* DCs were prepared according to Benestad.[6] Briefly, two DCs were filled with 0.1 ml Alpha Modified Essential Medium ($\alpha$-MEM) containing $5 \times 10^5$ murine bone marrow cells. These DCs were implanted in the peritoneal cavity of each host mouse 6 days after inflammation induction or sham operation, for a 3-day period. Then the DCs were removed and agitated for 1 h in $\alpha$-MEM containing 5% Ficoll and 0.5% pronase to dissolve any fibrin clot. The contents were harvested as described by Tyler *et al.*[7] Direct smears, differential counts and cell classification were performed as previously described.[8]

In the first part of the experiment, DCs were loaded with normal bone marrow cells and implanted in healthy and leukaemic mice, both challenged with aseptic abscesses, in order to examine the humoral regulatory system in each group. In the second part of the experiment, DCs loaded with leukaemic cells were implanted in healthy mice alone, with and without aseptic abscesses, in order to examine the leukaemic cell response to humoral regulators.

*Assay for colony stimulating activity:* Sera obtained from host mice were pooled and assayed for their ability to inhibit the growth of haemopoietic colonies in agar gel. Serum colony inhibitory activity was expressed by the reduction in the mean number of colonies produced by mixing 0.03 ml of tested serum with 0.13 ml horse serum when $10^5$ bone marrow cells were seeded. The number of granulocyte—monocyte colony forming units (CFU-GM) was determined in agar by a modified version that has been described previously.[9,10] Pokeweed mitogen-stimulated spleen cell conditioned medium (PWM-SCM) was prepared as previously described,[11] and added in all experiments to a final concentration of 20%. Colonies were counted on day 5 of culture. Three to five plates were counted for each experimental point.

*Statistics:* Results are expressed as means $\pm$ standard error of the means (SEM). Mann—Whitney's U test was used to assess the statistical significance between two comparable experimental groups, and the Kruskal—Wallis test was used to assess statistical significance between more than two groups.

### Results

*Diffusible granulopoietic inhibitor release in healthy and leukaemic mice:* Normal murine bone marrow cells were cultured in DCs implanted intraperitoneally both in normal and leukaemic hosts 6 days after copper insertion or sham operation. Three days after DC implantation we observed that, in healthy mice, DC cell growth was lower in the copper-inserted group than in the sham-operated mice (104.496 $\pm$ 11.157, 167.849 $\pm$ 14.608, respectively; $p < 0.025$, Figure 1). However, DC cell growth rate was not changed in leukaemic hosts with or without copper insertion (150.983 $\pm$ 11.890, 144.246 $\pm$ 12.309, respectively; $p > 0.2$, Figure 1). Moreover, the growth rate of DC granulocytes was significantly higher in leukaemic hosts when compared to healthy mice during the inhibition phase of acute inflammation ($p < 0.025$).

Changes in proliferative and non-proliferative granulocytes and in macrophages are shown in

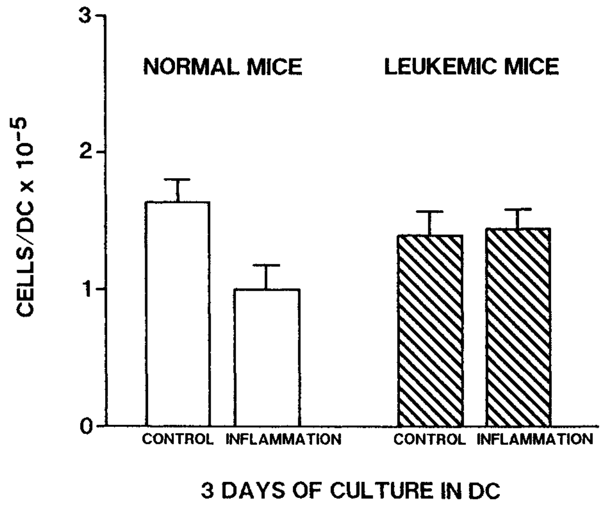

FIG. 1. The effect of acute inflammation inhibition phase on DC granulopoiesis in healthy and leukaemic hosts. Four groups of mice were studied: in each group (minimum three mice) at least six DCs were analysed for each experiment. The results are expressed as mean $\pm$ SEM for three experiments.

**Table 1.** The differential growth of normal murine bone marrow cells implanted into diffusion chambers. Cells were harvested on the third day of culture. The results represent mean values ± SEM from three experiments

| Host condition | Granulocyte/DC | | Macrophages |
|---|---|---|---|
| | Proliferative | Non-proliferative | |
| **Healthy mice** | | | |
| Sham-operated controls | 78.829 ± 7.614 | 38.216 ± 4.942 | 40.837 ± 6.878 |
| Copper-inserted mice | 48.842 ± 4.988 | 28.480 ± 3.844 | 27.342 ± 4.382 |
| **Leukaemic mice** | | | |
| Sham-operated controls | 68.432 ± 5.882 | 33.876 ± 4.128 | 31.488 ± 4.672 |
| Copper-inserted mice | 70.864 ± 7.134 | 36.684 ± 4.855 | 34.460 ± 4.825 |

Table 1. The production of both proliferative and non-proliferative granulocytes and of macrophages, is lower in DC implanted in copper-inserted healthy mice than in sham-operated controls ($p < 0.025$). However, in leukaemic copper-inserted hosts these numbers remained equal to those observed in sham-operated leukaemic controls ($p > 0.2$). These data indicate that humoral granulopoietic inhibitor release is demonstrable in healthy but not in leukaemic mice.

*Serum colony inhibitory activity in healthy and leukaemic mice:* Serum of sham-operated healthy controls permitted *in vitro* growth of 63 ± 9 CFU-GM, while serum of copper-inserted healthy mice during the inhibition phase of acute inflammation (6 days after copper insertion) permitted growth of 17 ± 5 CFU-GM. This statistically significant difference ($p < 0.01$) indicates that serum of copper-inserted mice contains an inhibitor to *in vitro* growth of granulocyte—monocyte colonies. However, in leukaemic hosts, copper insertion did not change serum levels of inhibitors ($p > 0.2$, Table 2).

*Leukaemic cell response to humoral inhibitors:* AML cells were implanted in DCs in healthy mice during the inhibition phase of acute inflammation. No change in DC leukocyte number was observed compared to the control ($p > 0.2$, Figure 2). This shows the lack of leukaemic cell response to humoral inhibitors. Moreover, the growth rate of leukaemic cells in DC was significantly higher than the growth rate of normal cells ($p < 0.01$), irrespective of prior inflammation. DC differential cell counts showed no change in maturation state of DC leukaemic cells implanted in copper-inserted mice. The number of blasts in DC implanted in copper-inserted mice was 156.432 ± 64.384, while the number of blasts in DC implanted in sham-operated hosts was 155.464 ± 48.212 ($p > 0.2$).

**Table 2.** Serum GM-colony growth inhibitor production in healthy and leukaemic mice during the inhibition phase of acute inflammation. Each value represents the mean ± SEM for three experiments. The serum was pooled, six days after the induction of inflammation, from five to eight mice in each group. Inhibitory activity was expressed as the mean number of CFU-GM colonies in agar gel.

| Host condition | CFU-GM/$10^5$ cultured bone marrow cells |
|---|---|
| **Healthy mice** | |
| Sham-operated controls | 63 ± 9 |
| Copper-inserted mice | 17 ± 5 |
| **Leukaemic mice** | |
| Sham-operated controls | 72 ± 11 |
| Copper-inserted mice | 74 ± 12 |

### Discussion

The induction of acute inflammation in healthy mice by subcutaneous insertion of sterile metallic copper rods, is associated with myeloid hyperplasia and a significant increase in the levels of serum colony stimulating factors (CSF), as well as an

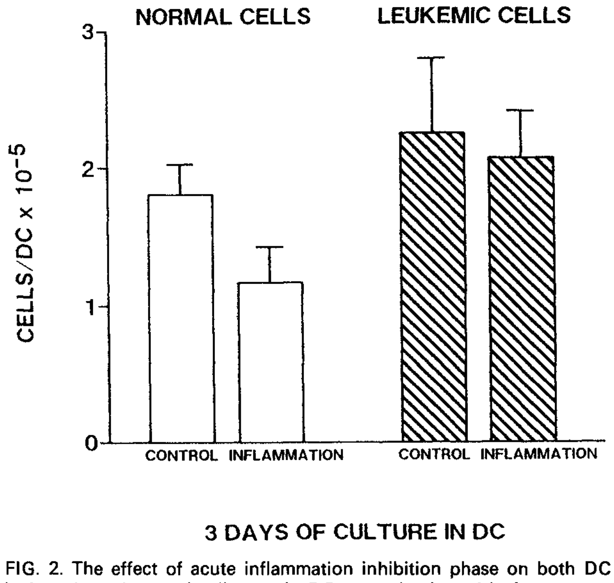

FIG. 2. The effect of acute inflammation inhibition phase on both DC leukaemic and normal cell growth. DCs were implanted in four groups of healthy mice: in each group (minimum three mice) at least six DCs were analysed for each experiment. The results are expressed as mean ± SEM for three experiments.

enhancement of DC granulopoiesis.[2,12,13] However, 6 days after induction of inflammation, the DC-granulopoiesis is inhibited, suggesting that diffusible granulopoietic humoral inhibitors are released,[2,14] and indicating a negative feedback control of *in vivo* granulopoiesis. Investigating the release of these humoral regulators in a leukaemic host is of special interest, as testing the capability of a leukaemic host to produce these factors might explain, in part, the mechanism of normal granulopoiesis failure in leukaemic subjects. Recent investigations[3] showed that leukaemic mice are unable to overproduce granulopoietic humoral stimulators during the stimulation phase of acute inflammation.

In this study we examined the humoral response of leukaemic mice during the inhibition phase of acute inflammation. The growth rate of normal DC-granulocytes implanted in leukaemic hosts proceeded to the same limiting concentrations with or without prior inflammation. This suggests that either leukaemic hosts are again unable to overproduce humoral granulopoietic inhibitors, or these inhibitors are functioning *in vivo* in an improper manner. However, several investigators have shown that leukaemic cells produce inhibitory substances which suppress normal granulopoiesis.[15,16] The possibility that the level of these inhibitory substances is almost maximum in leukaemic mice has been excluded, as the number of DC cells is even higher in leukaemic mice when compared to healthy mice during the inhibition phase.

*In vitro* growth of haemopoietic cells is stimulated by a group of glycoproteins, of different molecular weights, called the colony stimulating factors (CSF).[17] Our previous studies[12] showed that during the stimulation phase of acute inflammation the level of CSF is significantly high in the serum of normal mice. This is consistent with the increased levels of granulopoietic stimulators that enhance DC granulopoiesis in this phase of acute inflammation. In this study we investigated the presence of granulopoietic colony inhibitors in the serum of the host mice during the inhibition phase of acute inflammation. We observed that the serum of non-leukaemic mice contains inhibitory activity to granulocytic colony growth *in vitro*. This activity is not demonstrable in the serum of leukaemic mice challenged or non-challenged with inflammation. It is not known yet whether the inhibitory activity in the serum that acts on *in vitro* granulocytic growth, and the humoral granulopoietic inhibitor that depresses DC-granulocyte number *in vivo*, represents one or several factors. Their relationship to the previously described *in vitro* inhibitors, such as lactoferrin,[18] monocyte prostaglandin,[19] colony-stimulating activity inhibitors,[20] and the transforming growth factor beta (TGF-$\beta$),[21] is also unclear. However, both of them are produced in non-leukaemic mice but not in leukaemic ones during the inhibition phase. Recently, we showed that T-lymphocytes are involved in the release of granulopoietic humoral inhibitors, either directly or indirectly, by their interaction with other accessory bone marrow cells.[14] This raises the question of whether T-lymphocyte functional disorder in AML explains the lack of response in leukaemic mice during the inhibition phase of acute inflammation.

Unlike the growth rate of normal DC cells, the growth rate of leukaemic cells implanted into a non-leukaemic environment is unaffected by the rise in humoral inhibitor levels, as no change is observed in granulocyte cell proliferation and differentiation. The growth rate of leukaemic cells in DC implanted in normal mice, with or without prior inflammation, is significantly higher than the growth of normal cells. This shows that extrinsic physiological inhibitors are unable to reduce the growth rate of murine leukaemic cells, which proliferate irrespective of prior inflammation, reflecting uncontrolled proliferation of the leukaemic cells. These observations support the view that AML is the expression of an intrinsic cellular defect and is consistent with the findings of other investigators.[22] However, several studies showed that certain growth factors could inhibit leukaemic bone marrow cell growth *in vitro*, like TGF-$\beta$,[21] the leukaemia inhibitory factor,[23] and the granulocytic chalone.[24,25] Thus, it may be asked why leukaemic cells respond to *in vitro* inhibitors and not to *in vivo* humoral physiological inhibitors, but the answer is not clear.

However, the nature of *in vivo* inhibitors that have been released after the induction of inflammation, and their relation to the specific purified *in vitro* growth factors, is not known yet. They might be different regulatory factors, or their concentrations in *in vivo* normal physiological conditions may be lower than in *in vitro* exploited concentrations. Moreover, the colony stimulating factor receptor expression by AML blast cells has been shown to be weaker than that caused by the normal mature granulocytes.[26,27] This weakness in receptor expression could therefore be a possible explanation for the lack of leukaemic cell response to increased or decreased levels of physiological humoral factor regulators after inflammation induction.

Other models of diffusion chamber granulopoiesis enhancement have been investigated in hosts rendered neutropenic with myelotoxic drugs[7,28] or irradiation.[29] This has been interpreted as evidence for humoral influence on granulocyte production. Nevertheless, because of the numerous effects of myelotoxic drugs or irradiation, the changes leading to increased cell proliferation in DC might be quite nonspecific. Moreover, diffusion chamber granulopoiesis enhancement after endotoxin administration[30] will modify the host environment and can also affect directly the cellular content of DC. On the other hand, none of these models showed that granulopoiesis regulation in DC is under the influence of both humoral stimulators and inhibitors, as the aseptic abscess model showed. However, our findings are in agreement with a previous study[31] in which irradiation had been exploited as a stimulus to the growth of normal granulocytic cells in DC, since leukaemic cells proliferate equally well with or without prior host irradiation.

In conclusion, our findings show that leukaemic hosts are unable to respond properly to stimuli to release humoral granulopoietic inhibitors. They also show that leukaemic cells are not sensitive to increased levels of physiological humoral inhibitors, as neither their proliferation nor differentiation is affected.

### References

1. Niskanen E, Chatelain C, Symann M. Diffusion chamber colony-forming unit (CFU-d): a primitive stem cell. *Int J Cell Cloning* 1989; **7**: 330–342.
2. Symann M, Anckaert MA, Huybrecht M, Ninane J, Canon L, Sokal G. *In vivo* stimulation and inhibition of granulopoiesis: the effect of an inflammatory reaction on murine diffusion chamber granulopoiesis. *Br J Haematol* 1982; **15**: 89–98.
3. Hamood M, Corazza F, Bluche PF, El-Teraifi H, Fondu P. Acute inflammation effects on *in vivo* granulopoiesis: comparative studies in healthy and leukaemic mice. *In vivo* 1992; **6**: 45–48.
4. Major IR, Mole RH. Radiation-induced myeloid leukaemia in X-ray irradiated CBA mice. *Nature* 1978; **272**: 455–456.
5. Meldrum RA, Mole RH. Radiation-induced myeloid leukemia in CBA/H mice: a non-immunogenic malignant disease in syngeneic mice. *Br J Cancer* 1982; **45**: 403–412.
6. Benestad HB. Formation of granulocytes and macrophages in diffusion chamber cultures of mouse blood leukocytes. *Scand J Hematol* 1970; **7**: 279–288.
7. Tyler WS, Niskanen E, Stohlman F Jr, Keane J, Howard D. The effect of neutropenia on myeloid growth and the stem cell in an *in vivo* culture system. *Blood* 1972; **40**: 634–645.
8. Symann M, Quesenberry P, Fontebuoni A, Howard D, Ryan M, Stohlman F Jr. Fetal hemopoiesis in diffusion chamber culture III. The effect of neutropenia. *Blood* 1976; **48**: 283–291.
9. Bradley TR, Metcalf D. The growth of mouse bone marrow cells *in vitro*. *Aus J Exp Biol Med Sci* 1966; **44**: 287–299.
10. Chatelain C, Hamood M, Debast M, Symann M. Cholinergic enhancement of megakaryocytopoiesis and granulopoiesis in culture: mediation via T-lymphocytes. *Exp Hematology* 1989; **17**: 1067–1071.
11. Burstein SA, Adamson JW, Thoring DJ, Harker LA. Characteristics of murine megakaryocytic colonies *in vitro*. *Blood* 1979; **54**: 169–179.
12. Hamood M, Chatelain C, Fondu P, Symann M. *In vivo* elaboration of CSF in acute inflammation: proportionality to the intensity of the inflammatory stimulus and requirement of T-lymphocytes. *Eur J Hematol* 1990; **45**: 244–249.
13. Hamood M, Fondu P. *In vivo* regulation of granulocyte production in acute inflammation. *Cell Proliferation* 1991; **24**: 181–190.
14. Hamood M, Fondu P. *In vivo* inhibition of granulopoiesis in acute inflammation requires T-lymphocyte integrity. *Int J Cell Cloning* 1991; **9**: 134–143.
15. Broxmeyer HE, Jacobsen N, Kurland J, Mendelsohn N, Moore MA. *In vitro* suppression of normal granulocytic stem cells by inhibitory activity derived from human leukemia cells. *J Natl Cancer Inst* 1978; **60**: 497–511.
16. Logan PM, Shellard J, Buskard N, Levy JG. CAMAL: evidence for inhibitory role in normal granulopoiesis. *Exp Hematol* 1989; **17**: 1072–1076.
17. Burgess AW, Metcalf D. The nature and action of granulocyte—macrophage colony stimulating factors. *Blood* 1980; **56**: 947–958.
18. Broxmeyer HE, Smithyman A, Eger RR, Meyers PA, Desousa M. Identification of lactoferrin as the granulocyte-derived inhibitor of colony-stimulating activity production. *J Exp Med* 1978; **148**: 1052–1067.
19. Pelus LM, Broxmeyer HE, Kurland JI, Moore MAS. Regulation of macrophage and granulocyte proliferation. *J Exp Med* 1979; **150**: 277–292.
20. Verma DS, Spitzer G, Zander AR, Beran M, Dicke KA, McCredie KB. Monocyte—macrophage interaction with putative helper and suppressor T-lymphocyte in colony stimulating activity elaboration. In: Baum SJ, Ledney GD, eds. *Experimental Hematology Today* New York: Springer-Verlag, 1981; 139–150.
21. Sing GK, Keller JR, Ellingsworth LR, Ruscetti FW. Transforming growth factor $\beta$ selectively inhibits normal and leukemic human bone marrow cell growth *in vitro*. *Blood* 1988; **72**: 1504–1511.
22. Symann M, Rodhain J, Huybrechts C, *et al.* *In vivo* studies of a murine myeloblastic leukemia. *Nouv Rev Fr Hématol* 1980; **22**: 63–68.
23. Tomida M, Yamamoto-Yamaguchi Y, Hozumi M. Purification of a factor inducing differentiation of mouse myeloid leukemic M1 cells from conditioned medium of mouse fibroblast L929 cells. *J Biol Chem* 1984; **259**: 10978–10982.
24. Rytömaa T, Vilpo J, Levanto A, Jones WA. Effect of granulocytic chalone on acute myeloid leukemia in man. *Lancet* 1977; **1**: 771–774.
25. Foa P, Chillemi F, Lombardi L, Lonati S, Maiolo AT, Polli EE. Inhibitory activity of a synthetic pentapeptide in leukaemic myelopoiesis both *in vitro* and *in vivo* in rats. *Eur J Haematol* 1987; **39**: 399–403.
26. Budel LM, Touw IP, Delwel R, Clarck SC, Löwenberg B. Interleukin 3 (IL-3) and granulocyte—monocyte colony-stimulating factor receptors on human acute myelocytic leukemia cells and relationship to the proliferative response. *Blood* 1989; **74**: 565–571.
27. Motoji T, Watanabe M, Uzumaki H, *et al.* Granulocyte colony-stimulating factor (G-CSF) receptors on acute myeloblastic leukemia cells and their relationship with the proliferative response to G-CSF in clonogenic assay. *Br J Haematol* 1991; **77**: 54–59.
28. Niskanen E, Tyler WS, Symann M, Stohlman FJR, Howard D. The effect of neutropenia on the cell cycle of granulocyte precursors in an *in vivo* culture system. *Blood* 1974; **43**: 23–31.
29. Rothstein G, Hugl EH, Bishop CR, Athens JW, Ashenburger HE. Stimulation of granulopoiesis by a diffusible factor *in vivo*. *J Clin Invest* 1971; **50**: 2004–2007.
30. Rothstein G, Hugl EH, Chervenick PA, Athens JW, Macfarlane J. Humoral stimulators of granulocyte production. *Blood* 1973; **41**: 73–78.
31. Miller AM, Marmor JB, Page PL, Russell JL, Robinson SH. Unregulated growth of murine leukemic cells and suppression of normal granulocyte growth in diffusion chamber cultures. *Blood* 1976; **47**: 737–745.

ACKNOWLEDGEMENTS. The authors are indebted to M. De Bast and M. Roobaert for their technical assistance and to C. Vlietinck for preparation of the manuscript. M. Hamood is a research fellow of the Fonds National Belge de la Recherche Scientifique.

**Received 25 February 1992; accepted 25 March 1992**
